# Patterns of Genome-Wide VDR Locations

**DOI:** 10.1371/journal.pone.0096105

**Published:** 2014-04-30

**Authors:** Pauli Tuoresmäki, Sami Väisänen, Antonio Neme, Sami Heikkinen, Carsten Carlberg

**Affiliations:** Department of Biosciences, University of Eastern Finland, Kuopio, Finland; University of Tennessee, United States of America

## Abstract

The genome-wide analysis of the binding sites of the transcription factor vitamin D receptor (VDR) is essential for a global appreciation the physiological impact of the nuclear hormone 1α,25-dihydroxyvitamin D_3_ (1,25(OH)_2_D_3_). Genome-wide analysis of lipopolysaccharide (LPS)-polarized THP-1 human monocytic leukemia cells via chromatin immunoprecipitation sequencing (ChIP-seq) resulted in 1,318 high-confidence VDR binding sites, of which 789 and 364 occurred uniquely with and without 1,25(OH)_2_D_3_ stimulation, while only 165 were common. We re-analyzed five public VDR ChIP-seq datasets with identical peak calling settings (MACS, version 2) and found, using a novel consensus summit identification strategy, in total 23,409 non-overlapping VDR binding sites, 75% of which are unique within the six analyzed cellular models. LPS-differentiated THP-1 cells have 22% more genomic VDR locations than undifferentiated cells and both cell types display more overlap in their VDR locations than the other investigated cell types. In general, the intersection of VDR binding profiles of ligand-stimulated cells is higher than those of unstimulated cells. *De novo* binding site searches and HOMER screening for binding motifs formed by direct repeats spaced by three nucleotides (DR3) suggest for all six VDR ChIP-seq datasets that these sequences are found preferentially at highly ligand responsive VDR loci. Importantly, all VDR ChIP-seq datasets display the same relationship between the VDR occupancy and the percentage of DR3-type sequences below the peak summits. The comparative analysis of six VDR ChIP-seq datasets demonstrated that the mechanistic basis for the action of the VDR is independent of the cell type. Only the minority of genome-wide VDR binding sites contains a DR3-type sequence. Moreover, the total number of identified VDR binding sites in each ligand-stimulated cell line inversely correlates with the percentage of peak summits with DR3 sites.

## Introduction

The nuclear receptor VDR belongs to a transcription factor superfamily, members of which have the unique property to be directly activated by small lipophilic compounds [1]. Accordingly, the specific high-affinity ligand of VDR is the biologically most active vitamin D compound, 1,25(OH)_2_D_3_ [2]. The physiological impact of 1,25(OH)_2_D_3_ is not restricted to its well-known role in the homeostasis of calcium and phosphate being important for bone mineralization [3], but the nuclear hormone also has cell growth and immuno-modulatory functions [4], [5]. For example, in monocytes 1,25(OH)_2_D_3_ reduces the up-regulation of cytokines, such as tumor necrosis factor α and interleukins 1 and 6 [6], [7], i.e. VDR ligands can counteract pro-inflammatory signal transduction pathways, such as that of the transcription factor NF-κB [8]. Moreover, 1,25(OH)_2_D_3_ stimulation enhances the capacity of the immune system for anti-bacterial defense and to be more tolerogenic towards autoimmune phenomena [5]. Cells of the hematopoietic system, such as monocytes and macrophages, are important targets of 1,25(OH)_2_D_3_ [9], in which, for example, the expression of anti-bacterial proteins, such as cathelicidin antimicrobial peptide (CAMP), is promoted [10].

The current understanding of 1,25(OH)_2_D_3_ signaling suggests that genomic VDR binding sites and transcription start sites (TSSs) of the receptor's primary target genes need to share the same chromosomal domain [11]. In order to gain access to genomic DNA VDR has to compete with the intrinsic repressive nature of chromatin [12], [13]. *In vitro* studies have indicated that VDR preferentially binds to DR3-type sequences, which are preferentially bound by heterodimers of VDR with retinoid X receptor (RXR) [14], [15]. Non-liganded VDR can bind genomic DNA but then forms complexes with co-repressor proteins and histone deacetylases [16], [17]. In contrast, ligand-activated VDR changes its protein interaction profile to co-activator proteins and histone acetyltransferases [18]. Via mediator proteins ligand-activated VDR then contacts the basal transcriptional machinery, which is assembled on the TSS region, leading to transcriptional activation [12].

At present, VDR ChIP-seq data are available from i) GM10855 and GM10861 lymphoblastoid cells [19], ii) THP-1 monocyte-like cells [20], iii) LS180 colorectal cancer cells [21] and iv) LX2 hepatic stellate cells [22] reported 1,600-6,200 VDR-specific binding sites. These datasets have not yet been systematically compared, although there are first indications [23] that they show only a minor overlap, i.e. that the genome-wide binding of VDR is rather cell specific.

In studies of innate immunity and cancer, THP-1 cells are regularly used as a model system for 1,25(OH)_2_D_3_ signaling [20], [24]–[26]. The challenge of THP-1 cells with LPS, a constituent of the outer membrane of gram-negative bacteria, leads to a substantial change in their transcriptome profile, such as massive induction of pro-inflammatory cytokines, and polarizes them towards macrophages [27]. The phenotype of macrophages depends largely on the type of their stimulation [28]. Classically activated macrophages, such as after LPS stimulation, are defined as M1-type, while alternatively activated macrophages, such as those contributing to wound healing, are of M2-type. There are probably many intermediate states between these extremes, which all primarily differentiate via their cytokine expression profile.

In this study, we stimulated the M1-type polarized THP-1 cells with 1,25(OH)_2_D_3_ and determined the genome-wide VDR binding profile by ChIP-seq. In order to compare these new data with the five already published datasets, we re-analyzed the latter with the identical peak calling settings using Model-based Analysis for ChIP-seq (MACS), version 2, followed by a novel strategy to identify consensus summit locations across many samples. This meta-analysis of all available VDR ChIP-seq data concludes in a rather cell-specific profile of the receptor location. Moreover, the analysis demonstrated that the mechanistic basis for the action of the VDR is independent of the cell type. Furthermore, the number of identified VDR binding sites inversely correlates with the percentage of DR3-type sequences found below the ligand-stimulated peak summits.

## Materials and Methods

### Cell culture

The human acute monocytic leukemia cell line THP-1 [29] was grown in RPMI 1640 medium supplemented with 10% fetal calf serum, 2 mM L-glutamine, 0.1 mg/ml streptomycin and 100 U/ml penicillin and the cells were kept at 37 °C in a humidified 95% air/5% CO_2_ incubator. For polarization towards M1-type macrophages THP-1 cells were grown in a density of 500,000 cells/ml in phenol red-free RPMI 1640 medium supplemented with 5% charcoal-stripped fetal calf serum and incubated for 24 h with 100 ng/ml LPS (Sigma-Aldrich). Prior to chromatin extraction, the cells were exposed for 80 min with 10 nM 1,25(OH)_2_D_3_ or were left unstimulated.

### ChIP-seq and analysis

VDR ChIP-seq on the LPS-differentiated THP-1 cells was performed essentially as described before [20]. The ChIP templates were sequenced at 36 bp read length using standard manufacturer protocols at the Gene Core at the EMBL (Heidelberg, Germany). Alignment of sequence reads against the human reference genome version hg19 was done using Bowtie software version 1.0.0 [30] with the following essential command line arguments: bowtie -n 1 -m 1 -k 1 -e 70—best. In addition, the seed length (argument -l) was set individually for each sample as follows: 36 for LPS treated THP-1, THP-1, GM10855 and GM10861, 42 for LX2, 50 for LS180 stimulated lanes SRR342263, SRR342264 and unstimulated lane SRR342261 and 35 for rest of the LS180 samples. Of note, two read sets for the LPS-differentiated THP-1 samples (one unstimulated and one IgG) had an N as the 16th nucleotide to correct for a minor post-sequencing processing error at the sequencing platform, similar to some undifferentiated THP-1 read sets reported previously [20]. The short read data publically available under accession number GSE53041 contains the corrected reads. The read counts of all datasets are summarized in Table S4. The aligned input/IgG and VDR reads were converted to sorted BAM format using samtools [31] and, after merging the read sets per sample, converted to normalized TDF format using igvtools to allow efficient visualization in the IGV genome browser [32]. For ChIP-seq analysis statistically significant peaks were identified using MACS version 2 [33] with the following essential command line arguments: macs2 callpeak –bw 150 –keep-dup 1 –g hs —qvalue = 0.01 –m 5 50 —bdg. Otherwise, default parameters were used.

### Peak overlap analysis

**Figure 1:**
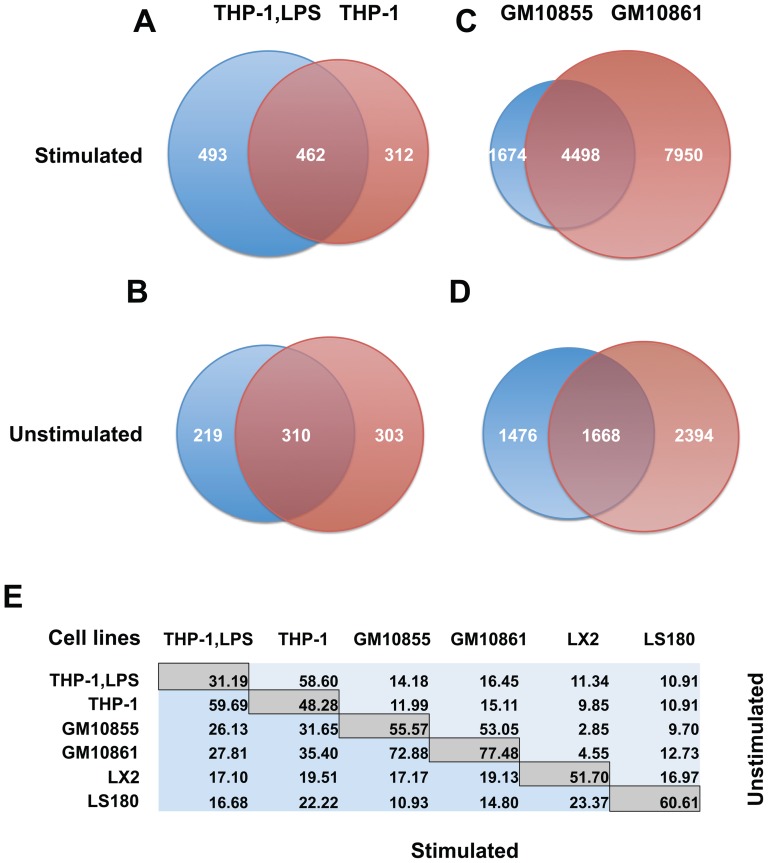
Overlap of VDR binding sites in stimulated and unstimulated cellular models. VDR ChIP-seq data from ligand-stimulated (**A**) and unstimulated (**B**) LPS-differentiated THP-1 cells (THP-1,LPS), as obtained in this study, were compared with respective data from undifferentiated THP-1 cells [20]. In the same way ligand-stimulated (**C**) and unstimulated (**D**) GM10855 and GM10861 cells [19] were matched. In the 'common' category of the peak counts both samples had a peak with a FDR <1%. When the narrower peak overlapped the wider by >50%, peaks were considered to be at the same location. **E** Summary of the % overlap of ligand-stimulated (blue) and unstimulated samples (light blue) of the four datasets used in A-D plus VDR ChIP-seq data from LX2 [22] and LS180 cells [21]. The percentages are indicated in reference to the sample with fewer peaks. A more detailed version of this figure can be found in the Supplements (Table S2).

For the pairwise comparisons (Fig. 1 and Table S2) peaks in the two samples were considered overlapping, if more than 50% of the narrower peak was overlapping the wider peak. The overlap percentages were defined as the proportion, in percent, of the peaks in the dataset with fewer peaks that overlapped with a peak in the dataset with more peaks. Table S2 also reports the percentages from the direction of the dataset with more peaks as well as the respective peak counts.

### DR3-type motif analysis

*De novo* motif search was done using HOMER [31] on ±100 bp peak summit regions for each of the 12 samples. Since the identified *de novo* motifs that HOMER matched to the reference DR3-type motif were very similar between the samples, we subsequently used only the motif for the stimulated, undifferentiated THP-1 sample as the representative DR3-type motif. This was mapped in peak sequences ±100 bp from the summit using the following essential command line arguments: annotatePeaks.pl <sample_name> hg19 –noann –nogene –m <motif_file> –size −100, 100.

### Finding consensus VDR ChIP-seq summit locations

The consensus VDR ChIP-seq summit locations presented in Table S1 were identified from MACS2 output peak data using custom R tools. In detail, all summit locations from all samples were extracted and analyzed using the R density function with triangular smoothing and bandwidth of 20. All densities under 1.0e^−15^ were zeroed to eliminate the internal noise of the R density function. Local density maxima, identified with a span of 100 bp, were considered as the consensus summits (with an accuracy of ±10 bp due to the density bandwidth). The relevant parameters of the summit density analysis are illustrated in the bottom panels of Fig. S5, where case A best depicts the accuracy and sensitivity. The consensus summits were then elongated 100 bp to both directions. Overlapping consensus peaks were eliminated by truncating the overlaps at their mid-point, resulting in peaks less than 200 bp in length. An example of overlapping initial consensus peaks is given in Fig. S5C. The borders and summit locations for the consensus peaks are reported in Table S1. Finally, to get necessary peak metrics also for those samples per consensus summit, which did not have an original MACS2 peak within the area of any consensus peak, we extracted the FEs and q-values from the score tracks generated by the MACS2 bdgcmp tool with the original MACS2 peak caller bedgraph outputs as input. Pseudocount of 1 was used when generating the FE score tracks.

### Functional characterization of VDR binding locations

The locations of all 23,409 VDR ChIP-seq peaks were analyzed by the Genomic Regions Enrichment of Annotations Tool (GREAT) [34], version 2.0.2. GREAT assigns biological meaning to a set of non-coding genomic regions by analyzing the functional annotations of the nearby genes. We applied the ‘basal plus extension’ mode assuming +/−20 kb as proximal and +/−400 kb as distal regions. From the results we focused on the ontology sets relating to biological pathways.

The summit regions (±100 bp) of all VDR ChIP-seq peaks were screened for DR3-type sequences by using HOMER with a minimal score of up to 4. The distance of these 9,058 DR3-type motifs to the closest single nucleotide polymorphism (SNP) of the genome-wide association study (GWAS) catalog [35] (www.genome.gov/gwastudies) is determined as the number of bases separating the central nucleotide of the DR3-type sequence and the location of the closest SNP. If the SNP is located upstream of the DR3-type sequence, the distance appears as negative.

## Results

### VDR binding profile in six cellular models

In order to obtain the genome-wide VDR binding profile of M1-type macrophages, we stimulated LPS-differentiated THP-1 cells with 1,25(OH)_2_D_3_ or left them untreated and then performed ChIP-seq with antibodies against VDR. The use of the peak calling software MACS, version 2, identified a total of 1,318 high-confidence VDR binding sites in this cell type (Table S1). In 1,25(OH)_2_D_3_-stimulated cells the number of VDR peaks was 953, while in unstimulated cells only 529 sites were found (Fig. S1A). The majority of the VDR binding sites in LPS-differentiated THP-1 cells, 789 (59.86%), were unique to stimulation with 1,25(OH)_2_D_3_, while 364 (27.62%) sites were identified specifically in unstimulated cells. This left only 165 (12.52%) VDR locations that were common in both stimulated and unstimulated cells.

Previously published VDR ChIP-seq datasets [19]–[22] had been analyzed with earlier versions of MACS or with other peak calling software. Moreover, the datasets from GM10855, GM10861, LS180 and LX2 cells [19], [21], [22] had originally been referenced to an earlier version of the human genome (hg18/NCBI36). Thus, in order to allow a direct comparison of the VDR location data, we re-analyzed all public VDR ChIP-seq datasets using MACS2 and referenced them to the latest version of the human genome (hg19). The dataset from lymphoblastoid cells [19] represents two immortalized B cell lines, GM10855 and GM10861, which originate from two individuals of the International HapMap Project [36]. We merged the respective VDR ChIP-seq duplicates but, in contrast to the original publication [19], kept the two cell lines separate to be able to investigate their potential differences (further details in the Methods).

An important finding obtained by our re-analysis is that the number of VDR binding sites differed substantially from the formerly reported counts. The dataset of undifferentiated THP-1 cells [20] reduced from the originally reported 2,340 to 1,080 VDR binding sites (471 (43.61%), 315 (29.17%) and 294 (27.22%) for unique stimulated, unique unstimulated and common, respectively; see also Fig. S1B). Originally, for the merged lymphoblastoid cell lines only 2,776 VDR peaks in 1,25(OH)_2_D_3_-stimulated and 623 in unstimulated cells had been reported, which is likely a result of the fairly stringent filtering strategy employed in that study [19]. In our analysis of the individual cell lines, the total number of VDR binding sites increased to 7,494 for GM10855 cells (4,350 (58.05%), 1,397 (18.64%) and 1,747 (23.31%) for unique stimulated, unique unstimulated and common, respectively; see also Fig. S1C) and even to 13,326 for GM10861 cells (9,254 (69.44%), 917 (6.88%) and 3,155 (23.68%) for unique stimulated, unique unstimulated and common, respectively; see also Fig. S1D). Finally, the total number of VDR binding sites in LX2 cells reduced from 6,281 [22] to 2,245 (771 (34.34%), 712 (31.71%) and 762 (33.94%) for unique stimulated, unique unstimulated and common, respectively; see also Table S2), while the VDR peak count in LS180 cells increased from 2,471 to 3,840 (3,675 (95.70%), 65 (1.69%) and 100 (2.60%) for unique stimulated, unique unstimulated and common, respectively; see also Table S2). When investigating the fraction of unstimulated peaks (for all cell lines, the smaller peak set) that overlapped the stimulated peaks, LPS-differentiated THP-1 cells interestingly showed, with only 31.19%, by far the lowest percentage of common VDR binding sites, while the percentage of unstimulated VDR peaks overlapping stimulated peaks in undifferentiated THP-1 cells was 48.28%, in LX2 cells 51.70%, in GM10855 cells 55.57%, in LS180 cells 60.61% and in GM10861 cells even 77.48% (Table S2).

In summary, genome-wide analysis of LPS-polarized THP-1 cells resulted in 1,318 high-confidence VDR binding sites, close to 60% of which are only observed after ligand stimulation. These represent 22% more genomic VDR locations than in undifferentiated THP-1 cells, but between 1.7- and 10-times less than in the other cellular models for which VDR ChIP-seq data are publically available.

### Cell-specificity of genome-wide VDR locations

The combined analysis of all six ChIP-seq datasets identified a set of 23,409 non-overlapping genomic VDR locations (Table S1). The vast majority of these, 17,700 (75.61%), are specific to one cell type, 4,945 (21.12%) are found in two, 469 (2.00%) in three, 179 (0.76%) in four, 73 (0.31%) in five and only 43 (0.18%) in all six cell types. Interestingly, when the maximally allowed distance between original peak summits was increased step-wise from 60 bp to 500 bp, the number of conserved VDR loci increased from 35 to 63, while in parallel the number of unique VDR binding sites reduced from 23,818 to 20,680 (Fig. S2). At the very short distances the likelihood of falsely separating summits that truly represent the same binding site is increased because the distance approaches the limit of accuracy of the ChIP-seq method itself. Conversely, longer distances will likely increase the number of falsely merged summits that are truly separate. We chose to use a fairly short 100 bp as the maximum allowed distance to mainly avoid falsely merged summits, but without excessive cost of falsely separated summits. Approximately 25% of all VDR locations therefore fit the hypothesis that conserved VDR binding is associated with more general functions of the receptor and its ligand 1,25(OH)_2_D_3_. The present dataset offers an opportunity to compare cell types with varying levels of relatedness. The two pairs, the LPS-differentiated and undifferentiated THP-1 cells as well as the two lymphoblastoid cell lines GM10855 and GM10861 are the best for a direct comparison of closely related cellular models (Fig. 1). Out of the 774 VDR binding sites observed in 1,25(OH)_2_D_3_-stimulated THP-1 cells, 462 (59.69%) were also found in stimulated LPS-differentiated THP-1 cells (Figs. 1A and E). A similar overlap (58.60% of the smaller dataset overlapping the larger) was also observed between these two cell types at the unstimulated state (Figs. 1B and E). For lymphoblastoid cells the corresponding percentages of overlapping VDR binding sites were 72.88% in stimulated samples (Figs. 1C and E) and 53.05% in unstimulated samples (Figs. 1D and E). Interestingly, the comparisons between the somewhat more distantly related cell types, i.e. those of lymphoblastic and monocytic origins, revealed intermediate overlaps, ranging from 26.13 to 35.40% in stimulated samples and 11.99 to 16.45% in unstimulated samples. In contrast, the overlaps in comparisons involving either LX2 or LS180 were lower, ranging from 10.93 to 23.37% for the stimulated samples and from 2.85 to 16.97% for the unstimulated samples (Fig. 1E). Collectively these pairwise comparisons across all the six cellular models (summary in Fig. 1E, for all data see Table S2) suggest that the VDR binding site overlap rate is higher when the cells are ligand-stimulated than when they are left unstimulated. The hierarchically clustered read accumulations ±1 kb around the total set of 23,409 VDR binding locations confirm both the similarity of closely related cell lines as well the difference of more distantly related cell lines (Fig. S3). A similar result was obtained when we used the webtool GREAT for analyzing the enrichment of genes in the vicinity of VDR loci for the PANTHER pathway ontology terms (Fig. S4).

Taken together, while the majority of genome-wide VDR binding sites are specific for one of the six analyzed cellular models, the rates of overlaps between the cell types follow roughly their developmental and functional relatedness. In general, the VDR binding profiles of ligand-stimulated cells show a higher level of overlap than those of unstimulated cells.

### VDR binding scenarios in LPS-polarized THP-1 cells

**Figure 2:**
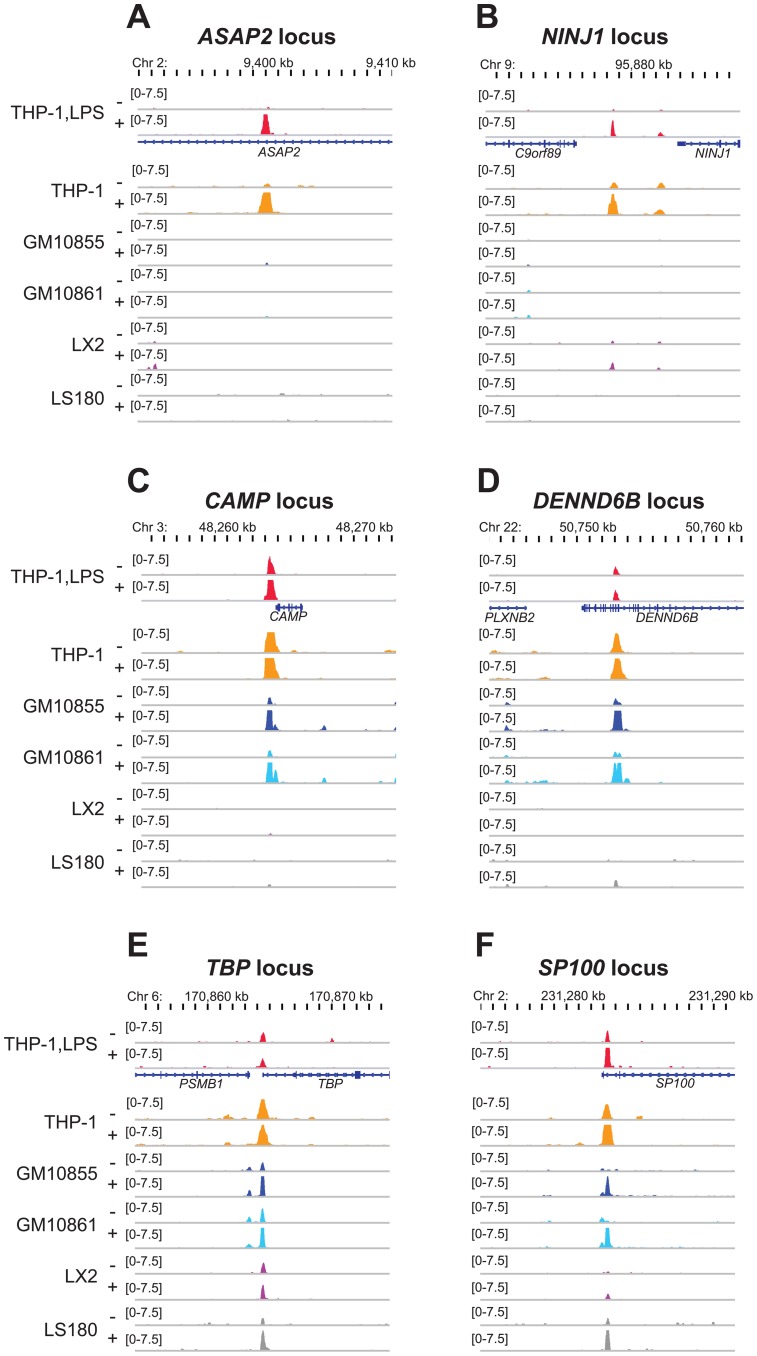
Different levels of cell type specificity of VDR peak locations. The IGV browser was used to display normalized VDR ChIP-seq signals in unstimulated (-) and ligand-stimulated (+) LPS-differentiated THP-1 cells (THP-1,LPS, red) for the loci of the genes *ASAP2* (**A**), *NINJ1* (**B**), *CAMP* (**C**), *DENND6B* (**D**), *TBP* (**E**) and *SP100* (**F**) in comparison to re-analyzed public data from undifferentiated THP-1 cells ([20], orange), the lymphoblastoid cell lines GM10855 ([19], dark blue) and GM10861 ([19], light blue), LX2 cells ([22], purple) and LS180 cells ([21], grey). Gene structures are indicated in blue.

From the 1,318 VDR binding sites in LPS-differentiated THP-1 cells (Table S1) 660 (50.08%) were observed specifically in this cell type (category 1). An example of this is the VDR binding site 160 kb downstream of the TSS of the gene prostaglandin E receptor 3 (*PTGER3*, Fig. S5A). At further 304 (23.07%) locations VDR binding was observed in one additional cell type (category 2). In accordance to the results described above (Fig. 1A) in the vast majority (77.96%) of cases the undifferentiated THP-1 cells were this second cell type. This is exemplified by a VDR binding site 54 kb downstream of the TSS of the gene ArfGAP with SH3 domain, ankyrin repeat and PH domain 2 (*ASAP2*, Fig. 2A), which we recently characterized as a primary VDR target [37]. At additional 134 (10.17%) genomic sites VDR was found in LPS-differentiated THP-1 cells and two further cell types (category 3). This is illustrated by VDR binding 18 kb downstream to the TSS of the ninjurin 1 (*NINJ1*) gene (Fig. 2B), which is known as a primary 1,25(OH)_2_D_3_ target in THP-1 cells [20]. A co-localization of VDR in LPS-differentiated THP-1 cells with three other cell types (category 4) was found for 117 (8.88%) genomic sites. This scenario is represented by a VDR binding site 0.5 kb upstream of the TSS of the well-established VDR target gene *CAMP* [10] (Fig. 2C). The constellation of VDR binding in LPS-differentiated THP-1 cells and four other cell types (category 5) was observed for only 60 loci (4.55%) and is monitored at the example of a site 12 kb downstream of the TSS of the gene DENN/MADD domain containing 6B (*DENND6B*, Fig. 2D). This gene, previously known as *FAM116*, is one of the most prominent primary 1,25(OH)_2_D_3_ targets in THP-1 cells [20]. Finally, genome-wide there are only 43 (3.26%) sites that are also targets of VDR in all of the other five cell types (category 6). Representative examples are sites i) 78 kb upstream of the trafficking protein, kinesin binding 1 (*TRAK1*) gene TSS (Fig. S5B), ii) at the TSS of the TATA box binding protein (*TBP*) gene (Fig. 2E) and iii) 0.5 kb downstream of the TSS of the SP100 nuclear antigen (*SP100*) gene (Fig. 2F). All three genes are known as primary targets of 1,25(OH)_2_D_3_ ([20] and unpublished results).

In summary, all these examples illustrate a multitude of scenarios where one to six cell types highlight the locations of VDR in relation to the TSS of its target genes. Importantly, only a very low number (43) of VDR loci are conserved between all six cell types.

### DR3-type sequences at genomic VDR locations

**Figure 3:**
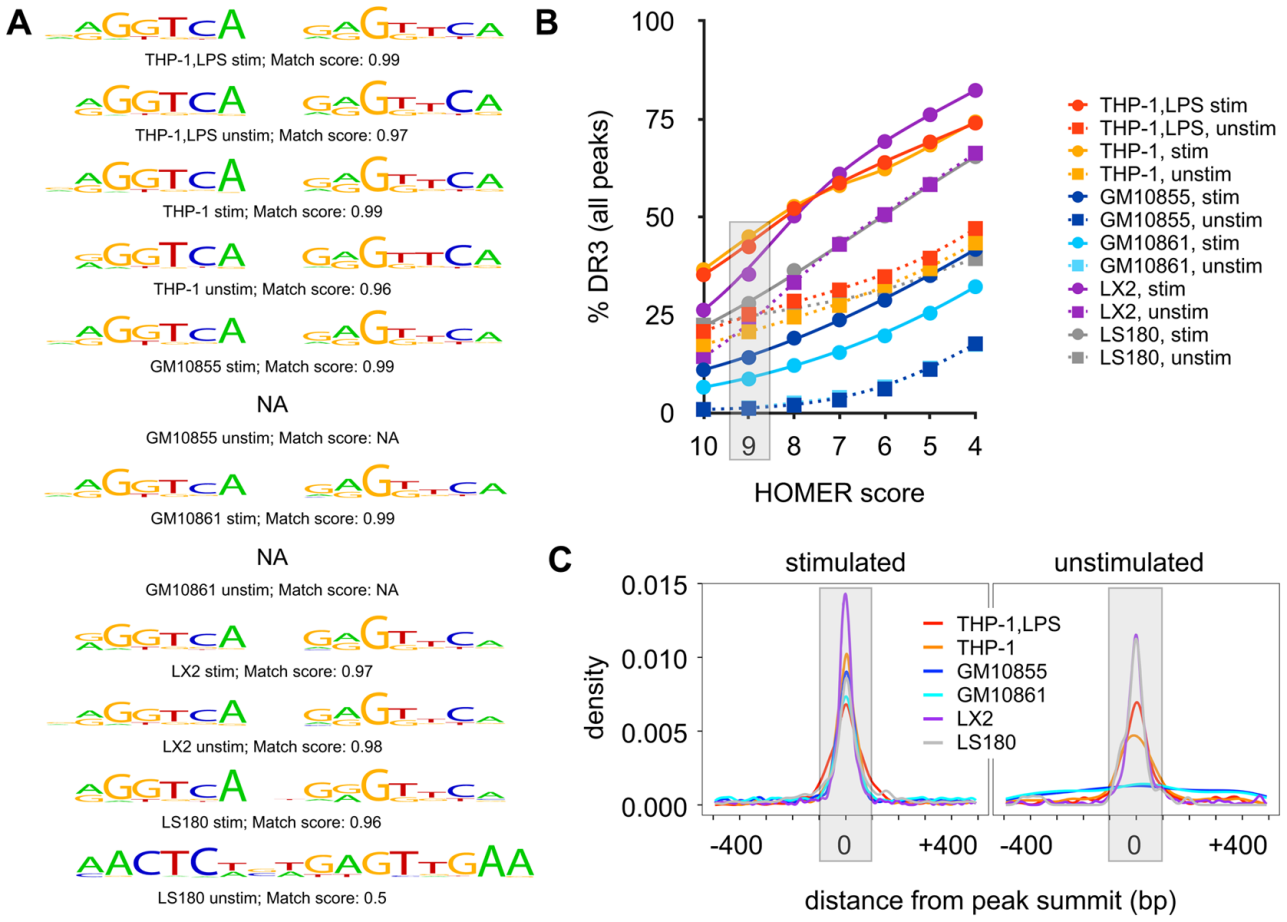
DR3-type sequences in VDR ChIP-seq peaks. **A** *De novo* analysis was performed on sequences below VDR peaks (±100 bp) of stimulated (stim) and unstimulated (unstim) datasets from LPS-differentiated THP-1 cells (THP-1,LPS), undifferentiated THP-1 cells, the lymphoblastoid cell lines GM10855 and GM10861, LX2 cells and LS180 cells. The sequence logos of DR3-type sequences are indicated, with the match score for the motif against the HOMER-native reference for the DR3-type sequence (range: 0–1). **B** HOMER was used, with variable thresholds, to screen for the representative DR3-type sequence, i.e. that from stimulated, undifferentiated THP-1 cells, in the same 12 datasets of sequences below VDR peaks. The percentage of identified DR3-type sequences are displayed as a function of the used HOMER scores. The grey box highlights the HOMER score for the representative DR3-type sequence (9.184643) that was used for the plot in Fig. 4 and Table S1. **C** The density of the representative DR3-type sequence is displayed ±500 bp around the peak summits. For clarity, the stimulated (left panel) and unstimulated (right panel) samples are displayed separately. The gray boxes delineate the ±100 bp region around the peak summits.

In order to obtain mechanistic information from the total of 23,409 VDR binding sites identified across all samples (Table S1), we first performed *de novo* binding site searches of the sequences below the VDR peak summits (±100 bp, Fig. 3A) using the HOMER tool set [38]. Nine of the 12 investigated VDR peak sets (six cellular models, ligand-stimulated and unstimulated) provided the same result, which is the classical DR3-type consensus sequence **RGGTCA**NNG**RGTTCA** for VDR-RXR heterodimers [14], [15]. In addition, the rather small set of 165 VDR peaks in unstimulated LS180 cells suggested a longer motif, but also this resembled a DR3-type sequence. In contrast, for the rather large sets of 3,144 and 4,072 VDR peaks of unstimulated GM10855 and GM10861 cells, respectively, our *de novo* search did not indicate any DR3-type sequence (Fig. 3A).

In addition to *de novo* binding site searches, we screened for DR3-type sequences below VDR ChIP-seq peak summits, defining the search area as ±100 bp around the peak summit (Figs. 3B and C). The HOMER score indicates the cumulative log-odds probability for a given sequence to match the searched motif, i.e. the lower the score, the poorer the match. To become identified as a valid match by HOMER, the score for a given sequence must exceed the motif-specific score threshold that maximized, upon the original *de novo* identification, its enrichment in the peak sequences over background sequences. Unlike HOMER, however, some motif screening algorithms do not indicate clear general or motif-specific thresholds for the detection of the significant matches. We therefore tested the effect of manually selected thresholds on the identification of DR3-type sequences by using as representative *de novo* DR3-like motif the one identified in ligand-stimulated THP-1 cells that has the HOMER-native, optimized score threshold of 9.184643. As expected, the percentage of peaks with a DR3-type sequence increased upon lowering the threshold, but even with the lowest tested HOMER score of 4, remained below 85% for any cell line (Fig. 3B). However, only 9,058 (38.7%) of the full set of 23,409 consensus VDR peaks carry a DR3-type motif at that low stringency of a HOMER score of 4 (Table S3). We screened these 9,058 DR3-type sequences for the closest SNP contained in the GWAS catalog [35] and found two that overlap with a DR3 motif (Table S3). These were i) rs6590322 that has been associated with Alzheimer's disease [39] and is located in the spacer of a DR3 motif of a VDR loci some 250 kb downstream of the TSS of gene for the transcription factor ETS1, and ii) rs10174949 that is related to allergies [40] and is found in the third position of the second half-site of the VDR binding site located 26 kb downstream of the TSS of the long non-coding RNA 299 gene.

At the HOMER-native threshold for the representative DR3-type motif, the 12 datasets showed a very similar ranking in the percentage of DR3-type sequence as observed already by the *de novo* searches. In ligand-stimulated, undifferentiated THP-1 cells 44.96% of the sequences below VDR peak summits contained a high-confidence DR3-type sequence, while only 20.69% of the peaks in unstimulated cells had such a site. The respective percentages for LPS-differentiated THP-1 cells are 42.39 and 25.14%, for LX2 cells 35.38 and 21.44%, for LS180 cells 27.96 and 24.85%, for GM10855 cells 14.13 and 1.15% and for GM10861 cells 8.66 and 1.28% (Fig. 3B). Of note, the very low percentage of DR3-type motifs in the unstimulated lymphoblastoid cells is the likely reason why the *de novo* search failed to identify any such motif in these relatively large peak sets (see Fig. 3A).

In order to verify that the DR3-type sequences were concentrated in the immediate peak summit region, we also screened for the DR3-type sequences within a wider, 1 kb region centered at the VDR ChIP-seq peak summits. We found that indeed the DR3-type sequences were highly enriched to approximately ±100 bp region surrounding the peak summits in all samples except the two unstimulated lymphoblastoid peak sets that have very few DR3-type sequences to begin with (Fig. 3C). Therefore, all VDR ChIP-seq peaks that only have DR3-type sequences outside the central ±100 bp region may result from some other binding mode than direct DNA-binding to a consensus sequence as a VDR-RXR heterodimer.

The lack of DR3-type sequences in a considerable proportion of the VDR peak summit regions in all analyzed samples led us to search for alternative sequences that can be identified below VDR peaks using HOMER. Performing the analysis separately on peak sets that did or did not contain a DR3 motif within ±100 bp of summit was designed to suggest transcription factors that may i) co-occur with VDR when it is bound to the DR3 motif, i.e. as a VDR-RXR heterodimer, and, importantly, ii) partner with VDR in the other binding modes. As expected, the DR3-type sequence ranked the first in all 12 datasets that *a priori* contained it (Fig. S6A). All peak sets of the monocytic origin as well as the stimulated lymphoblastoid peak sets shared binding sites for the transcription factors PU.1 (also called SPI1), ESRRB (also called NR3B2) and GABPA as the next most significantly enriched motifs. For LX2, the highest ranked non-DR3-sequence was the motif for the transcription factor JUN (being part of the AP1 complex), followed by ESRRB for stimulated peaks and GABPA for both stimulated and unstimulated peaks. In stimulated LS180 cells, the top non-DR3-sequences were the motifs for the transcription factors SMAD, REVERB (also called NR1D1) and JUN. The unstimulated peak sets for the two lymphoblastoid cell lines as well as LS180 were considered too small to provide reliable results.

For the peak sets, which are *a priori* without a DR3-type motif (Fig. S6B), the stimulated monocytic cell lines shared motifs for the transcription factors CEBP and PU.1, while the unstimulated monocytic cell lines shared most highly ranked motifs for the transcription factors NFYA, LHX3-like and NANOG. A considerable exception was the top-most ranking of NFYA in the stimulated, undifferentiated THP-1 cells, whereas NFYA was not within the top 4 of most significantly enriched motifs in the stimulated, LPS-differentiated THP-1 cells. In lymphoblastoid cells the motif for the transcription factor GABPA was the top ranked motif in all four samples, followed by those for the transcription factor BORIS (also called CTCF-like), with the exception of stimulated GM10855 cells, where BORIS was replaced by IRF4. In stimulated lymphoblastoid cells also PU.1-like motifs ranked high, whereas in unstimulated lymphoblastoid cells motifs for the transcription factor complex GFI1-STAF did the same. In contrast, both samples of LX2 cells had motifs for the transcription factors JUN, TEAD4 and RUNX as the top ranked. The stimulated LS180 cells also had JUN as the top ranking motif, but unlike for LX2 cells it had HNF4A (also called NR2A1) and GFI1-STAF motifs as the next best ranked. In the small peak set for the unstimulated LS180 cells, motifs for the transcription factors STAT5, CHR and ZFX ranked the highest.

Taken together, our *de novo* searches and DR3-type binding site screenings demonstrated as common findings for all six datasets that i) ligand-stimulated samples show a higher rate of DR3-type sequences than unstimulated samples of the same cellular model, ii) the more VDR peaks a stimulated dataset contains, the lower is the percentage of its DR3-type sequences, iii) by far not all VDR binding sites even at the stimulated state contain a DR3-type sequence and iv) the occurrence of non-DR3-type motifs differ considerably between the cell lines, especially in peaks that lack DR3-type motifs.

### VDR ChIP-seq peak quality relates to DR3-type binding site percentage

**Figure 4:**
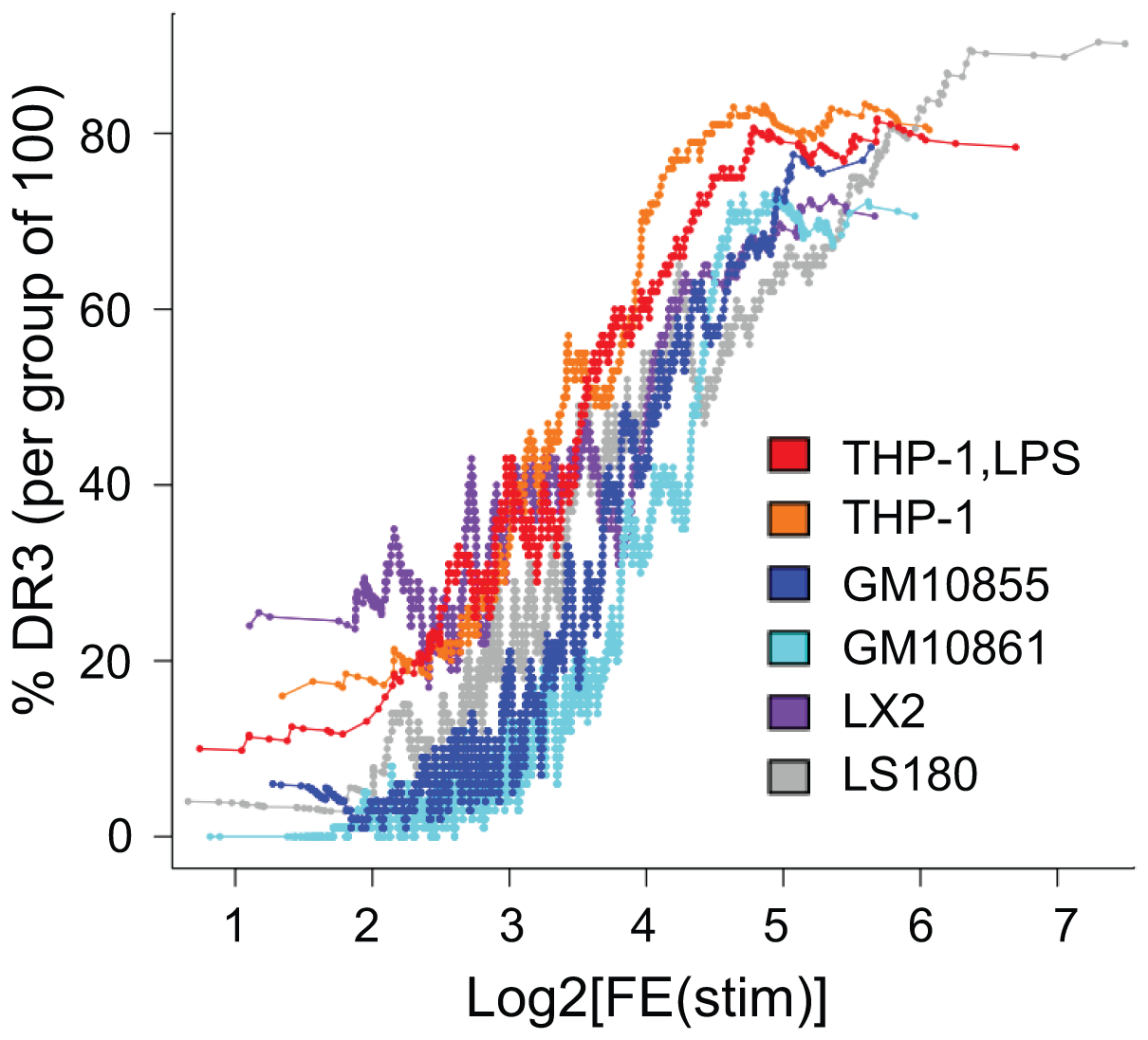
Correlation between DR3 percentage and VDR peak size. The VDR peaks of the ChIP-seq datasets of ligand-stimulated LPS-differentiated THP-1 cells (THP-1,LPS, red), undifferentiated THP-1 cells (orange), the lymphoblastoid cell lines GM10855 (dark blue) and GM10861 (light blue), LX2 cells (purple) and LS180 cells (grey) were individually ranked by their FE-value. For each set of 100 consecutively ranked peaks the average DR3 percentage (HOMER score >9.184643) was calculated and plotted as a function of the FE-value.

Next we asked whether the fold enrichment (FE) values of the VDR ChIP-seq peaks, are related to the percentage of DR3-type sequences found below the peaks. Therefore, we ranked the datasets of ligand-stimulated cells (Table S1) by their FE value and determined the percentage of peak locations with a DR3-type sequence (HOMER score >9.184643) in each set of 100 consecutively ranked peaks (Fig. 4). Each of the six VDR ChIP-seq datasets provided a similar sigmoidal curve as a result: peaks with low FE showed a low DR3 percentage, but the more the FE value increased, the more frequently the DR3-type sequences were found below the VDR peaks, up to a saturation level well below 100%. Upon detailed inspection, however, the individual DR3 percentage curves showed some minor cell-specific differences: i) the curves for LPS-differentiated and undifferentiated THP-1 cells start with a DR3 percentage of above 10%, rise quickly and reach saturation at 80%, ii) the curve for LX2 cells begins at above 20%, shows a less steep incline compared to THP-1 cells and meets saturation already at 70%, iii) the curve for LS180 cells opens below 5%, increases even less steeply, but finally results in a saturation at more than 90% and iv) the curves for GM10855 and GM10861 cells start at very low DR3 rates (6 and 0%), rise the least steeply and reach saturation already at a DR3-type sequence percentage of 70%.

In summary, we made the observation that the six VDR ChIP-seq datasets display essentially the same relation between the VDR peak size and the percentage of DR3-type sequences below the peak summits.

## Discussion

The genome-wide location of VDR is an essential information for understanding the pleiotropic physiological action of 1,25(OH)_2_D_3_. In this study, we generated VDR ChIP-seq data for LPS-differentiated THP-1 cells. These cells resemble M1-type macrophages and represent another tissue that is important for the interpretation of the immune-modulatory function of 1,25(OH)_2_D_3_. This new VDR ChIP-seq dataset was compared with all the publically available VDR ChIP-seq datasets that we re-analyzed using identical settings and taking the benefit of the recent advances in the relevant bioinformatic tools. Thus, this study also represents the first meta-analysis of VDR ChIP-seq data from six different cell types and sets the basis for a compendium of all VDR binding sites genome-wide.

The six ChIP-seq datasets differ largely in their total number of genome-wide VDR binding sites. While the two THP-1 datasets (LPS-differentiated and undifferentiated) provide only 1,100-1,300 genome-wide VDR locations, the two lymphoblastoid cell lines suggest an up to 10-times higher number. This difference could arise from the number of sequence tags obtained in the ChIP-seq procedure, differences in signal-to-noise ratios or variations in the reference sample (IgG or input) or simply from a much higher VDR expression in B lymphocytes than in monocytes/macrophages. However, the more likely explanation is that in B cells more VDR binding sites are accessible, i.e. the level of epigenomics. Nevertheless, it can be questioned, whether the regulation of a few hundred primary VDR target genes per tissue requires a far higher number of high quality genomic VDR binding sites. Although the two lymphoblastoid cell lines provided the highest number of VDR binding sites, they scored by far the lowest for the percentage of DR3-type sequences below the VDR peak summits. Therefore, the total number of VDR binding sites, which ChIP-seq identifies in a given cell type, is not a reliable indication on the quality of the respective dataset.

As expected, the VDR binding profile of LPS-differentiated THP-1 cells resembles the most that of undifferentiated THP-1 cells. Nevertheless, 50% of the 1,318 VDR binding sites in LPS-differentiated THP-1 cells are unique to this cellular model. However, this is still a low percentage, since for the total of 23,409 non-overlapping VDR binding sites a full 75% are observed only in one cell type. These unique VDR binding sites may be the mediators of cell-type specific actions of the receptor and its ligand. In fact, on the level of VDR target gene expression, as measured by microarrays [41]–[44], it is already known that in most tissues a rather different set of genes respond to stimulation with 1,25(OH)_2_D_3_. On the other hand, VDR locations that overlap between two or more tissues represent independent confirmations of the validity of a VDR binding site. Moreover, genomic regions that are recognized in multiple cell types by VDR may have a more generalized, and therefore likely higher impact on the physiological actions of the receptor and its ligand than the cell type specific sites. For example, the response of the *CAMP* gene to 1,25(OH)_2_D_3_ in many hematopoietic cell types is probably of larger impact on the function of the immune system than the specific response of the *PTGER3* gene in LPS-differentiated THP-1 cells. Approximately half of the few tens of VDR binding sites that are conserved in all investigated cell types are located close to TSS regions, i.e. in genomic loci that are more likely within open chromatin than other areas of the genome. Therefore, these sites may indicate preferential entry points for the VDR to the genome from which, probably via 3-dimensional network interactions, other more distal 1,25(OH)_2_D_3_-responsive regions are controlled.

VDR peaks that contain a consensus DR3-type sequences below their summits are assumed to function via classical VDR-RXR heterodimers, which has been characterized in numerous *in vitro* examples [45]–[47]. In this study, we demonstrated that in all six ChIP-seq datasets of ligand-stimulated cell types the size of the VDR peaks is associated with a high percentage of DR3-type consensus sequences below their summits. Moreover, *de novo* binding site searches in these six datasets resulted in the same DR3-type consensus sequence. Of note, at our default settings of a HOMER score of 9.18, only 2,686 (11.47%) out of the total of 23,409 VDR binding sites carry a DR3-type sequence, although this is mostly due to the lymphoblastoid cell lines that had the highest number of peaks but the lowest percentage of DR3-type sequences. The DR3 rate of 47.32% for ligand-stimulated LPS-differentiated THP-1 cells is, on the other hand, one of the highest within the present set of cell lines, and comparable to that of undifferentiated THP-1 cells.

A less stringent definition of the DR3-type consensus sequence, i.e. considering sequences with a lower HOMER score, or inclusion of hits immediately proximal to the peak summit, or both, can considerably increase the DR3 rate of a dataset. The ranking of the cell types for their DR3 frequency was largely independent of the chosen threshold for the specificity of the DR3-type sequence. However, this should not be taken as generally applicable, because it is largely the product of our systematic and uniform analysis. Moreover, even assuming a lower binding specificity of VDR-RXR heterodimers, on average less than 50% of VDR peaks contain a DR3-type binding site. This suggests that in every 1,25(OH)_2_D_3_-responsive cell type VDR is found not only in classical VDR-RXR heterodimers binding DR3-type sequences but also in alternative protein-DNA complexes. The latter contain a larger variety of transcription factors, such as GABPA, JUN, NFY, PU.1 and STAT5. The selection for these alternative VDR partners may largely depend on the cell-specific expression levels of these proteins.

In all ligand-stimulated samples the rate of DR3-type sequences is higher than in the respective unstimulated samples, i.e. the latter contain a higher rate of non-DR3-type VDR binding sites. Although also unliganded VDR is functional, for example actively repressing some target genes via the interaction with co-repressor proteins [16], [48], a number of the VDR binding sites in unstimulated cells may serve as lower affinity nuclear storage sites from where the receptor, upon ligand stimulation, moves to the binding sites of activated genes as we already hypothesized earlier [20].

In conclusion, a new VDR ChIP-seq dataset from M1-type macrophage-like cells was the initiation for performing the first meta-analysis of all publicly available VDR ChIP-seq datasets. In contrast to general expectations, only some 11.5% of all 23,409 VDR binding sites contain a DR3-type sequence. Moreover, the number of identified VDR binding sites inversely correlates with the percentage of DR3-type sequences found below the peak summits. This context allowed a better understanding of the VDR binding pattern in LPS-differentiated THP-1 cells, which with 1,318 VDR peaks is rather small, but shows for ligand-stimulated samples a DR3 rate of close to 50%.

## Availability of Supporting Data

The datasets supporting the results of this article are available at GEO (www.ncbi.nlm.nih.gov/geo) under the accession number GSE53041.

## Supporting Information

Figure S1 VDR binding site overlap between stimulated and unstimulated samples of four hematopoietic models. VDR ChIP-seq peak counts from LPS-differentiated THP-1 cells (THP-1,LPS, this study, **A**), from undifferentiated THP-1 cells ([20], **B**) and from the lymphoblastoid cell lines GM10855 (**C**) and GM10861 ([19], **D**) are presented for locations of ligand-stimulated (blue) and unstimulated categories (red). In the 'common' category of the peak counts both samples had a peak with a FDR <1%. When the narrower peak overlapped the wider by >50%, peaks were considered to be at the same location.
(PDF)

Figure S2 The effect of maximal allowed peak summit distance on the number of conserved and total VDR ChIP-seq peaks. The VDR ChIP-seq peak overlap analysis was repeated by varying the maximal allowed peak summit distance over the indicated distances. The number of genomic loci conserved in all six datasets was plotted over the distance (red). The inset shows the total number of unique VDR peaks over the same distances (blue).
(PDF)

Figure S3 Hierarchical clustering of VDR ChIP-seq signals. The densities of aligned VDR ChIP-seq reads ±1 kb around each of the 23,409 consensus peak summits for all 12 datasets were hierarchically clustered and plotted using the ngsplot.r tool (http://code.google.com/p/ngsplot). Each dataset is individually scaled to normalize for differences in the total read counts. The intensity of the red color indicates the relative amount of reads aligned to the region.
(PDF)

Figure S4 Hierarchical clustering of GREAT analysis of VDR ChIP-seq peak loci. The web-tool GREAT was used to analyze the genes in the vicinity of the six VDR ChIP-seq datasets of ligand-stimulated cells for specific enrichment in ontology terms in the PANTHER pathway classification system (www.pantherdb.org/pathway). Pathways with adjusted p-value <0.05 in any of the six cell lines were hierarchically clustered and plotted as a heat map using the –log10-transformed adjusted p-values.
(PDF)

Figure S5 Consensus peak definitions. The IGV browser was used to display exemplary scenarios of VDR ChIP-seq peak summits in unstimulated (-) and ligand-stimulated (+) LPS-differentiated THP-1 cells (THP-1,LPS, red) in comparison to re-analyzed public data from undifferentiated THP-1 cells ([20], orange), the lymphoblastoid cell lines GM10855 ([19], dark blue) and GM10861 ([19], light blue), LX2 cells ([22], purple) and LS180 cells ([21], grey). Gene structures are indicated in blue. The bottom panels depict how the consensus summit assignment strategy resolves the exemplary scenarios for case of **A**) a single peak summit in a single cell line (closest gene: *PTGER3*), **B**) single peak summits in many cell lines (closest gene: *TRAK1*) and **C**) several nearby peak summits that are variably present in several cell lines (closest gene: *ARID1A*). For the bottom panels, the black line shows the triangular density of all summits in the region, the horizontal dashed green line indicates the density cutoff (10−15) used to remove the effective zero densities, the vertical red line indicates the positions of original MACS2 peak summits, the vertical blue line marks the position of the identified consensus summit, and the vertical dashed orange lines indicate the positions of consensus peak borders before eliminating the overlaps.
(PDF)

Figure S6 The occurrence of known transcription factor binding motifs below VDR ChIP-seq peaks. Known binding motifs were screened in the ±100 bp VDR ChIP-seq peak sets with (**A**) or without (**B**) the representative *de novo* DR3-type motif, ranked according to the significance of motif enrichment, subset to include only those that were within the top 4 in any sample, and displayed as hierarchically clustered heatmap of the ranks. Prior to screening, the known motif set supplied with the HOMER version 4.3 was reduced to remove similar motifs using a similarity cutoff 0.8 and replacing the HOMER-native DR3-type motif by the representative DR3-type motif from this study, i.e. that from stimulated, undifferentiated THP-1 cells. The numbers of screened peaks are given below the heatmaps for each sample. The + and - sign after the sample names on top indicate, respectively, whether the sample was ligand stimulated or not.
(PDF)

Table S1 VDR ChIP-seq peak locations. The border and summit locations of the 23,409 VDR ChIP-seq peaks obtained from ligand-stimulated (stim) and unstimulated (unstim) LPS-differentiated THP-1 cells (THP-1,LPS), undifferentiated THP-1 cells [20], the lymphoblastoid cell lines GM10855 and GM10861 [19], LX2 cells [22] and LS180 cells [21] are indicated. Genomic locations that are displayed in Figs. 2 and S4 are highlighted in red. Moreover, FE and fold change (FC) values are provided for each VDR location and cellular model. The DR3-type sequences below the summits of the peaks were determined by HOMER (score >9.184643) and the location of the binding site relative to the peak summit is indicated.
(XLSX)

Table S2 Overlap of VDR binding sites in stimulated and unstimulated cellular models. VDR ChIP-seq data from LPS-differentiated THP-1 cells (THP-1,LPS) were compared with respective data from undifferentiated THP-1 cells [20], the lymphoblastoid cell lines GM10855 and GM10861 cells [19], LX2 cells [22] and LS180 cells [21]. Peak counts are given for both categories and the % overlap was calculated from larger to smaller samples (red) and from smaller to larger samples (green). Only the latter are summarized in Fig. 1E.
(XLSX)

Table S3 Overlap of DR3-type VDR binding sites with GWAS SNPs. The genomic regions (±100 bp) below all 23,409 VDR ChIP-seq peaks were analyzed by using HOMER with a minimal score of 4 (i.e. lowest stringency) for the presence of a DR3-type sequence. The position and sequence of these 9,058 DR3-type motifs is indicated as well as their distance to the closest SNP of the GWAS catalog [35]. Overlap of DR3-type VDR binding sites with GWAS SNPs. The genomic regions (±100 bp) below all 23,409 VDR ChIP-seq peaks were analyzed by using HOMER with a minimal score of 4 (i.e. lowest stringency) for the presence of a DR3-type sequence. The position and sequence of these 9,058 DR3-type motifs is indicated as well as their distance to the closest SNP of the GWAS catalog [35].
(XLSX)

Table S4 Read and peak counts for VDR ChIP-seq datasets.
(XLSX)

## References

[1] Perissi V, Rosenfeld MG (2005) Controlling nuclear receptors: the circular logic of cofactor cycles. Nat Rev Mol Cell Biol 6: 542–554. 15957004 10.1038/nrm1680

[2] Carlberg C, Molnár F (2012) Current status of vitamin D signaling and its therapeutic applications. Curr Top Med Chem 12: 528–547. 22242854 10.2174/156802612799436623

[3] DeLuca HF (2004) Overview of general physiologic features and functions of vitamin D. Am J Clin Nutr 80: 1689S–1696S. 15585789 10.1093/ajcn/80.6.1689S

[4] Ingraham BA, Bragdon B, Nohe A (2008) Molecular basis of the potential of vitamin D to prevent cancer. Curr Med Res Opin 24: 139–149. 18034918 10.1185/030079908x253519

[5] Verstuyf A, Carmeliet G, Bouillon R, Mathieu C (2010) Vitamin D: a pleiotropic hormone. Kidney Int 78: 140–145. 20182414 10.1038/ki.2010.17

[6] Giulietti A, van Etten E, Overbergh L, Stoffels K, Bouillon R, et al (2007) Monocytes from type 2 diabetic patients have a pro-inflammatory profile. 1,25-Dihydroxyvitamin D_3_ works as anti-inflammatory. Diabetes Res Clin Pract 77: 47–57. 17112620 10.1016/j.diabres.2006.10.007

[7] Prehn JL, Fagan DL, Jordan SC, Adams JS (1992) Potentiation of lipopolysaccharide-induced tumor necrosis factor-alpha expression by 1,25-dihydroxyvitamin D_3_ . Blood 80: 2811–2816. 1450407

[8] Yu XP, Bellido T, Manolagas SC (1995) Down-regulation of NF-kappa B protein levels in activated human lymphocytes by 1,25-dihydroxyvitamin D_3_ . Proc Natl Acad Sci U S A 92: 10990–10994. 7479923 10.1073/pnas.92.24.10990 PMC40556

[9] Hewison M (2011) Antibacterial effects of vitamin D. Nat Rev Endocrinol 7: 337–345. 21263449 10.1038/nrendo.2010.226

[10] Gombart AF, Borregaard N, Koeffler HP (2005) Human cathelicidin antimicrobial peptide (CAMP) gene is a direct target of the vitamin D receptor and is strongly up-regulated in myeloid cells by 1,25-dihydroxyvitamin D_3_ . Faseb J 19: 1067–1077. 15985530 10.1096/fj.04-3284com

[11] Carlberg C, Campbell MJ (2013) Vitamin D receptor signaling mechanisms: Integrated actions of a well-defined transcription factor. Steroids 78: 127–136. 23178257 10.1016/j.steroids.2012.10.019 PMC4668715

[12] Carlberg C, Seuter S (2010) Dynamics of nuclear receptor target gene regulation. Chromosoma 119: 479–484. 20625907 10.1007/s00412-010-0283-8 PMC2938416

[13] Eberharter A, Becker PB (2002) Histone acetylation: a switch between repressive and permissive chromatin. Second in review series on chromatin dynamics. EMBO Rep 3: 224–229. 11882541 10.1093/embo-reports/kvf053 PMC1084017

[14] Umesono K, Murakami KK, Thompson CC, Evans RM (1991) Direct repeats as selective response elements for the thyroid hormone, retinoic acid, and vitamin D_3_ receptors. Cell 65: 1255–1266. 1648450 10.1016/0092-8674(91)90020-y PMC6159884

[15] Carlberg C, Bendik I, Wyss A, Meier E, Sturzenbecker LJ, et al (1993) Two nuclear signalling pathways for vitamin D. Nature 361: 657–660. 8382345 10.1038/361657a0

[16] Polly P, Herdick M, Moehren U, Baniahmad A, Heinzel T, et al (2000) VDR-Alien: a novel, DNA-selective vitamin D_3_ receptor-corepressor partnership. Faseb J 14: 1455–1463. 10877839 10.1096/fj.14.10.1455

[17] Malinen M, Saramäki A, Ropponen A, Degenhardt T, Väisänen S, et al (2008) Distinct HDACs regulate the transcriptional response of human cyclin-dependent kinase inhibitor genes to trichostatin A and 1α,25-dihydroxyvitamin D_3_ . Nucleic Acids Res 36: 121–132. 17999998 10.1093/nar/gkm913 PMC2248733

[18] Gronemeyer H, Gustafsson JA, Laudet V (2004) Principles for modulation of the nuclear receptor superfamily. Nat Rev Drug Discov 3: 950–964. 15520817 10.1038/nrd1551

[19] Ramagopalan SV, Heger A, Berlanga AJ, Maugeri NJ, Lincoln MR, et al (2010) A ChIP-seq defined genome-wide map of vitamin D receptor binding: associations with disease and evolution. Genome Res 20: 1352–1360. 20736230 10.1101/gr.107920.110 PMC2945184

[20] Heikkinen S, Väisänen S, Pehkonen P, Seuter S, Benes V, et al (2011) Nuclear hormone 1α,25-dihydroxyvitamin D_3_ elicits a genome-wide shift in the locations of VDR chromatin occupancy. Nucleic Acids Res 39: 9181–9193. 21846776 10.1093/nar/gkr654 PMC3241659

[21] Meyer MB, Goetsch PD, Pike JW (2012) VDR/RXR and TCF4/beta-catenin cistromes in colonic cells of colorectal tumor origin: impact on c-FOS and c-MYC gene expression. Mol Endocrinol 26: 37–51. 22108803 10.1210/me.2011-1109 PMC3248320

[22] Ding N, Yu RT, Subramaniam N, Sherman MH, Wilson C, et al (2013) A vitamin D receptor/SMAD genomic circuit gates hepatic fibrotic response. Cell 153: 601–613. 23622244 10.1016/j.cell.2013.03.028 PMC3673534

[23] Carlberg C, Seuter S, Heikkinen S (2012) The first genome-wide view of vitamin D receptor locations and their mechanistic implications. Anticancer Res 32: 271–282. 22213316

[24] Gynther P, Toropainen S, Matilainen JM, Seuter S, Carlberg C, et al (2011) Mechanism of 1α,25-dihydroxyvitamin D_3_-dependent repression of interleukin-12B. Biochim Biophys Acta 1813: 810–818. 21310195 10.1016/j.bbamcr.2011.01.037

[25] Matilainen JM, Husso T, Toropainen S, Seuter S, Turunen MP, et al (2010) Primary effect of 1α,25(OH)_2_D_3_ on IL-10 expression in monocytes is short-term down-regulation. Biochim Biophys Acta 1803: 1276–1286. 20691220 10.1016/j.bbamcr.2010.07.009

[26] FANTOM-Consortium, Suzuki H, Forrest AR, van Nimwegen E, Daub CO, et al (2009) The transcriptional network that controls growth arrest and differentiation in a human myeloid leukemia cell line. Nat Genet 41: 553–562. 19377474 10.1038/ng.375 PMC6711855

[27] Sharif O, Bolshakov VN, Raines S, Newham P, Perkins ND (2007) Transcriptional profiling of the LPS induced NF-kappaB response in macrophages. BMC Immunol 8: 1. 17222336 10.1186/1471-2172-8-1 PMC1781469

[28] Murray PJ, Wynn TA (2011) Protective and pathogenic functions of macrophage subsets. Nat Rev Immunol 11: 723–737. 21997792 10.1038/nri3073 PMC3422549

[29] Tsuchiya S, Kobayashi Y, Goto Y, Okumura S, Nakae S, et al (1982) Induction of maturation in culture human monocytic leukemia cells by phorbol diester. Cancer Res 42: 1530–1536. 6949641

[30] Langmead B, Trapnell C, Pop M, Salzberg SL (2009) Ultrafast and memory-efficient alignment of short DNA sequences to the human genome. Genome Biol 10: R25. 19261174 10.1186/gb-2009-10-3-r25 PMC2690996

[31] Li H, Handsaker B, Wysoker A, Fennell T, Ruan J, et al (2009) The Sequence Alignment/Map format and SAMtools. Bioinformatics 25: 2078–2079. 19505943 10.1093/bioinformatics/btp352 PMC2723002

[32] Robinson JT, Thorvaldsdottir H, Winckler W, Guttman M, Lander ES, et al (2011) Integrative genomics viewer. Nat Biotechnol 29: 24–26. 21221095 10.1038/nbt.1754 PMC3346182

[33] Zhang Y, Liu T, Meyer CA, Eeckhoute J, Johnson DS, et al (2008) Model-based analysis of ChIP-Seq (MACS). Genome Biol 9: R137. 18798982 10.1186/gb-2008-9-9-r137 PMC2592715

[34] McLean CY, Bristor D, Hiller M, Clarke SL, Schaar BT, et al (2010) GREAT improves functional interpretation of cis-regulatory regions. Nat Biotechnol 28: 495–501. 20436461 10.1038/nbt.1630 PMC4840234

[35] Welter D, MacArthur J, Morales J, Burdett T, Hall P, et al (2014) The NHGRI GWAS Catalog, a curated resource of SNP-trait associations. Nucleic Acids Res 42: D1001–1006. 24316577 10.1093/nar/gkt1229 PMC3965119

[36] HapMap-Consortium (2003) The International HapMap Project. Nature 426: 789–796. 14685227 10.1038/nature02168

[37] Seuter S, Ryynanen J, Carlberg C (2013) The ASAP2 gene is a primary target of 1,25-dihydroxyvitamin D in human monocytes and macrophages. J Steroid Biochem Mol Biol. 10.1016/j.jsbmb.2013.08.014 23999061

[38] Heinz S, Benner C, Spann N, Bertolino E, Lin YC, et al (2010) Simple combinations of lineage-determining transcription factors prime cis-regulatory elements required for macrophage and B cell identities. Mol Cell 38: 576–589. 20513432 10.1016/j.molcel.2010.05.004 PMC2898526

[39] Potkin SG, Guffanti G, Lakatos A, Turner JA, Kruggel F, et al (2009) Hippocampal atrophy as a quantitative trait in a genome-wide association study identifying novel susceptibility genes for Alzheimer's disease. PLoS One 4: e6501. 19668339 10.1371/journal.pone.0006501 PMC2719581

[40] Hinds DA, McMahon G, Kiefer AK, Do CB, Eriksson N, et al (2013) A genome-wide association meta-analysis of self-reported allergy identifies shared and allergy-specific susceptibility loci. Nat Genet 45: 907–911. 23817569 10.1038/ng.2686 PMC3753407

[41] Palmer HG, Sanchez-Carbayo M, Ordonez-Moran P, Larriba MJ, Cordon-Cardo C, et al (2003) Genetic signatures of differentiation induced by 1α,25-dihydroxyvitamin D_3_ in human colon cancer cells. Cancer Res 63: 7799–7806. 14633706

[42] Wang TT, Tavera-Mendoza LE, Laperriere D, Libby E, MacLeod NB, et al (2005) Large-scale in silico and microarray-based identification of direct 1,25-dihydroxyvitamin D_3_ target genes. Mol Endocrinol 19: 2685–2695. 16002434 10.1210/me.2005-0106

[43] Suzuki T, Tazoe H, Taguchi K, Koyama Y, Ichikawa H, et al (2006) DNA microarray analysis of changes in gene expression induced by 1,25-dihydroxyvitamin D_3_ in human promyelocytic leukemia HL-60 cells. Biomed Res 27: 99–109. 16847355 10.2220/biomedres.27.99

[44] White SL, Belov L, Barber N, Hodgkin PD, Christopherson RI (2005) Immunophenotypic changes induced on human HL60 leukaemia cells by 1α,25-dihydroxyvitamin D_3_ and 12-O-tetradecanoyl phorbol-13-acetate. Leuk Res 29: 1141–1151. 16111532 10.1016/j.leukres.2005.02.012

[45] Toell A, Polly P, Carlberg C (2000) All natural DR3-type vitamin D response elements show a similar functionality in vitro. Biochem J 352: 301–309. 11085922 PMC1221460

[46] Orlov I, Rochel N, Moras D, Klaholz BP (2012) Structure of the full human RXR/VDR nuclear receptor heterodimer complex with its DR3 target DNA. EMBO J 31: 291–300. 22179700 10.1038/emboj.2011.445 PMC3261568

[47] Schräder M, Nayeri S, Kahlen JP, Müller KM, Carlberg C (1995) Natural vitamin D_3_ response elements formed by inverted palindromes: polarity-directed ligand sensitivity of vitamin D_3_ receptor-retinoid X receptor heterodimer-mediated transactivation. Mol Cell Biol 15: 1154–1161. 7862109 10.1128/mcb.15.3.1154 PMC230337

[48] Sanchez-Martinez R, Zambrano A, Castillo AI, Aranda A (2008) Vitamin D-dependent recruitment of corepressors to vitamin D/retinoid X receptor heterodimers. Mol Cell Biol 28: 3817–3829. 18362166 10.1128/MCB.01909-07 PMC2423301

