# Supplementary figures and images for: Patterns of Genome-Wide VDR Locations
Source: PLoS One. 2014 Apr 30;9(4):e96105. doi: 10.1371/journal.pone.0096105 (PMC4005760; doi:10.1371/journal.pone.0096105)

**Fig. S1**

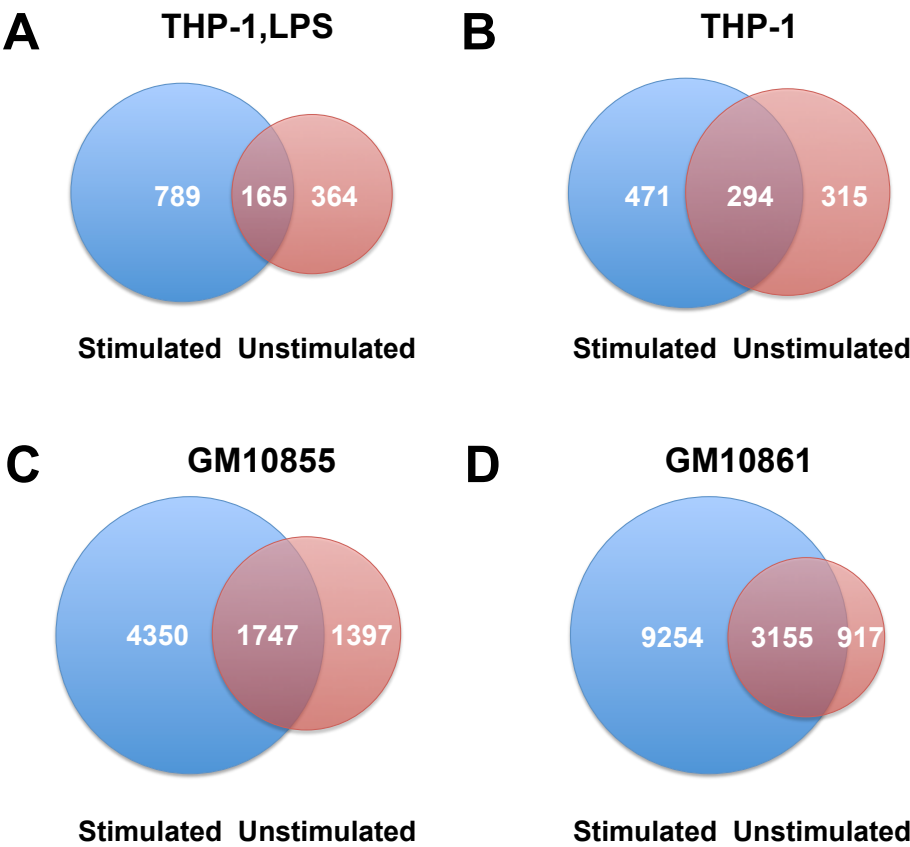

Supplement: Figure S1 — VDR binding site overlap between stimulated and unstimulated samples of four hematopoietic models. VDR ChIP-seq peak counts from LPS-differentiated THP-1 cells (THP-1,LPS, this study, A), from undifferentiated THP-1 cells ([20], B) and from the lymphoblastoid cell lines GM10855 (C) and GM10861 ([19], D) are presented for locations of ligand-stimulated (blue) and unstimulated categories (red). In the 'common' category of the peak counts both samples had a peak with a FDR <1%. When the narrower peak overlapped the wider by >50%, peaks were considered to be at the same location. (PDF) [file pone.0096105.s001.pdf]

**Fig. S2**

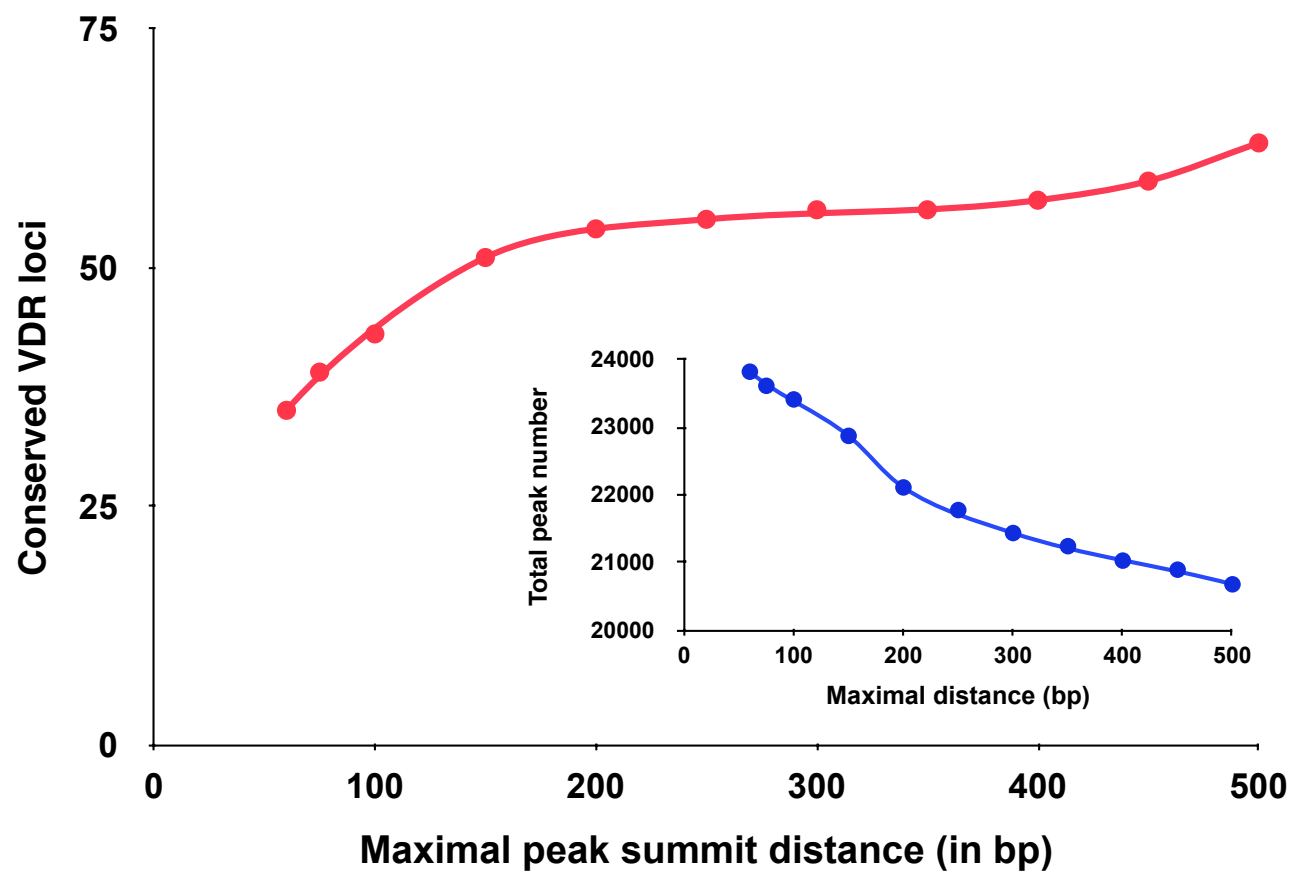

Supplement: Figure S2 — The effect of maximal allowed peak summit distance on the number of conserved and total VDR ChIP-seq peaks. The VDR ChIP-seq peak overlap analysis was repeated by varying the maximal allowed peak summit distance over the indicated distances. The number of genomic loci conserved in all six datasets was plotted over the distance (red). The inset shows the total number of unique VDR peaks over the same distances (blue). (PDF) [file pone.0096105.s002.pdf]

Fig. S3

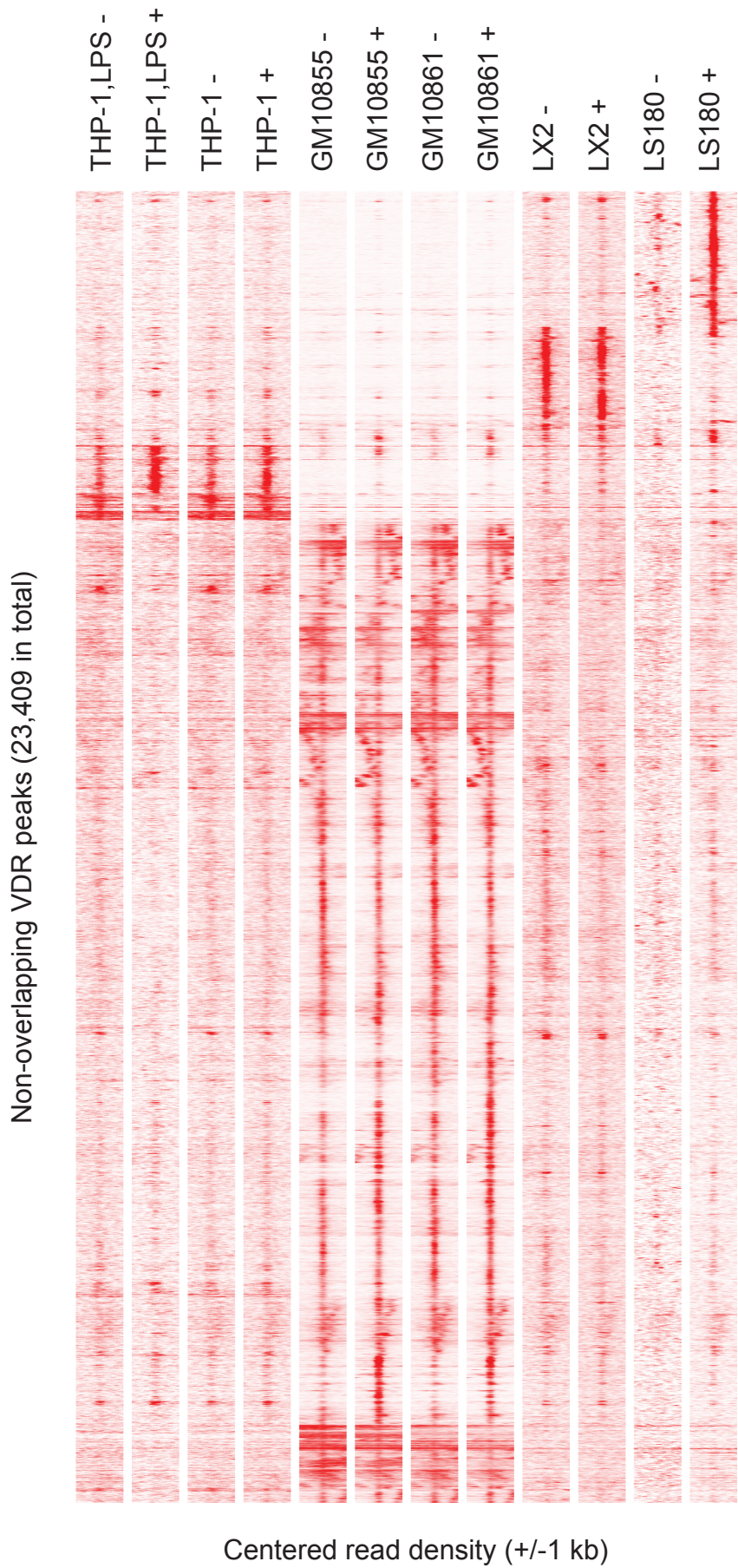

Supplement: Figure S3 — Hierarchical clustering of VDR ChIP-seq signals. The densities of aligned VDR ChIP-seq reads ±1 kb around each of the 23,409 consensus peak summits for all 12 datasets were hierarchically clustered and plotted using the ngsplot.r tool (http://code.google.com/p/ngsplot). Each dataset is individually scaled to normalize for differences in the total read counts. The intensity of the red color indicates the relative amount of reads aligned to the region. (PDF) [file pone.0096105.s003.pdf]

# Fig. S4

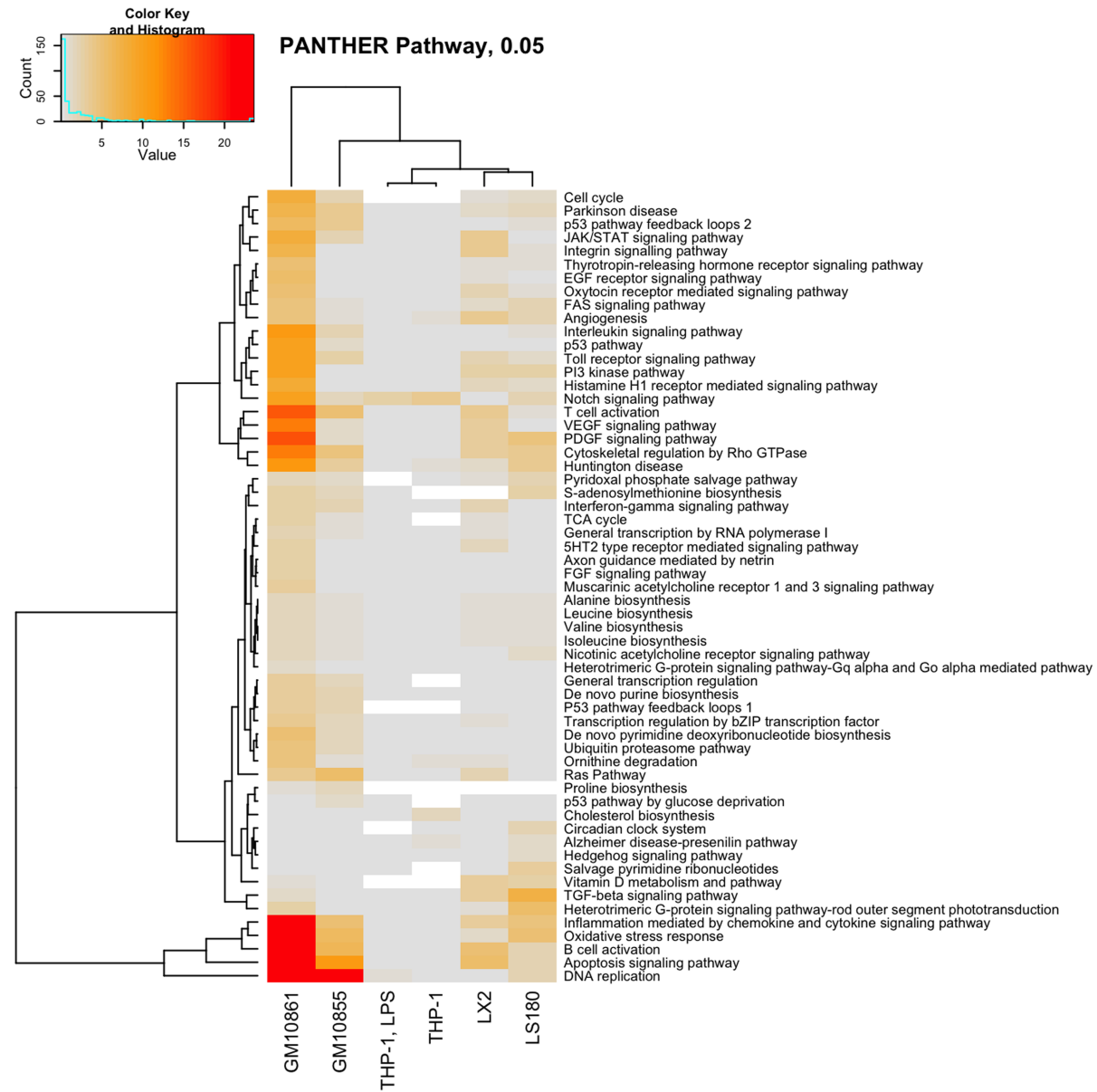

Supplement: Figure S4 — Hierarchical clustering of GREAT analysis of VDR ChIP-seq peak loci. The web-tool GREAT was used to analyze the genes in the vicinity of the six VDR ChIP-seq datasets of ligand-stimulated cells for specific enrichment in ontology terms in the PANTHER pathway classification system (www.pantherdb.org/pathway). Pathways with adjusted p-value <0.05 in any of the six cell lines were hierarchically clustered and plotted as a heat map using the –log10-transformed adjusted p-values. (PDF) [file pone.0096105.s004.pdf]

Fig. S5

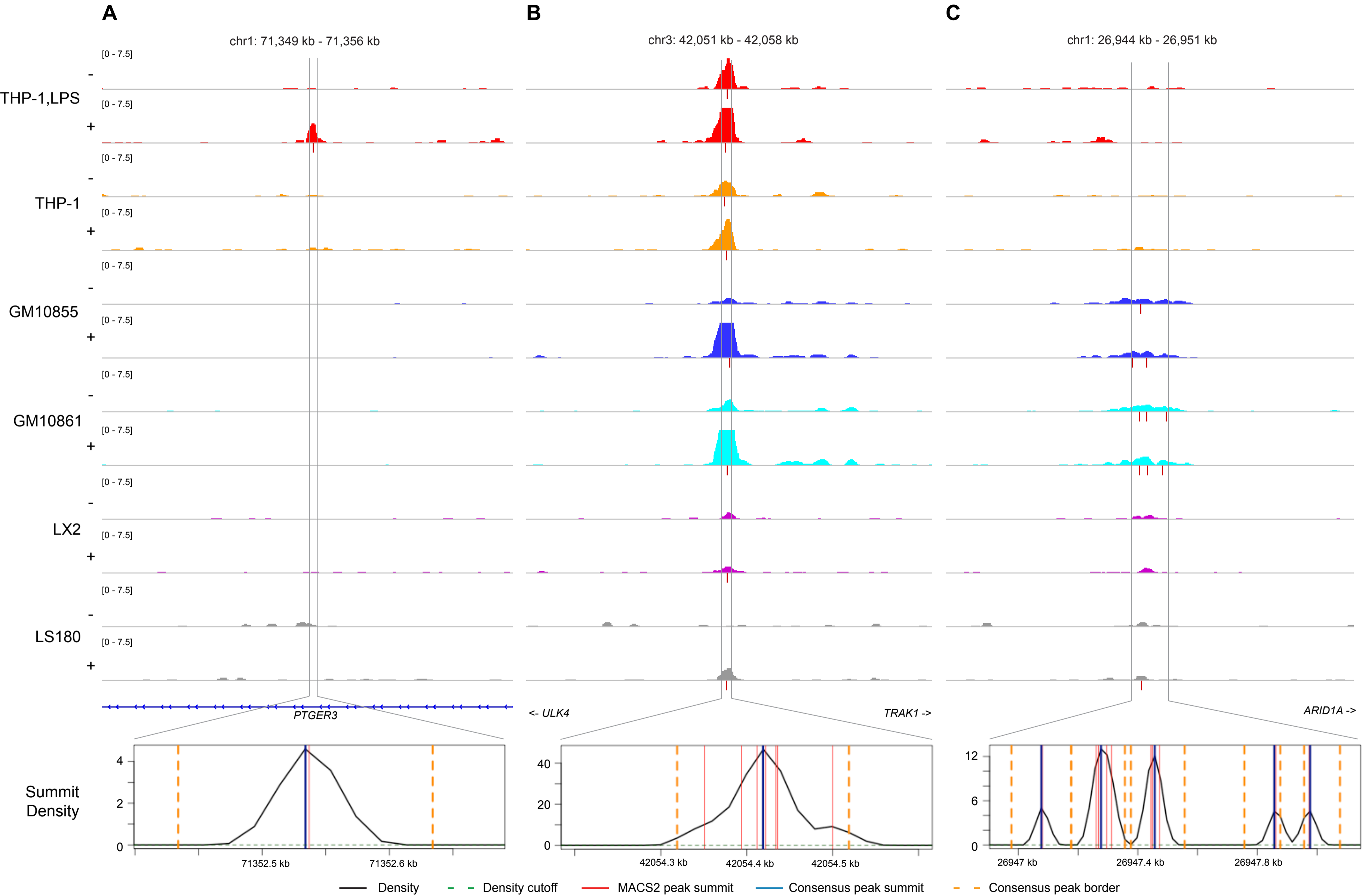

Supplement: Figure S5 — Consensus peak definitions. The IGV browser was used to display exemplary scenarios of VDR ChIP-seq peak summits in unstimulated (-) and ligand-stimulated (+) LPS-differentiated THP-1 cells (THP-1,LPS, red) in comparison to re-analyzed public data from undifferentiated THP-1 cells ([20], orange), the lymphoblastoid cell lines GM10855 ([19], dark blue) and GM10861 ([19], light blue), LX2 cells ([22], purple) and LS180 cells ([21], grey). Gene structures are indicated in blue. The bottom panels depict how the consensus summit assignment strategy resolves the exemplary scenarios for case of A) a single peak summit in a single cell line (closest gene: PTGER3), B) single peak summits in many cell lines (closest gene: TRAK1) and C) several nearby peak summits that are variably present in several cell lines (closest gene: ARID1A). For the bottom panels, the black line shows the triangular density of all summits in the region, the horizontal dashed green line indicates the density cutoff (10−15) used to remove the effective zero densities, the vertical red line indicates the positions of original MACS2 peak summits, the vertical blue line marks the position of the identified consensus summit, and the vertical dashed orange lines indicate the positions of consensus peak borders before eliminating the overlaps. (PDF) [file pone.0096105.s005.pdf]

Fig. S6

A

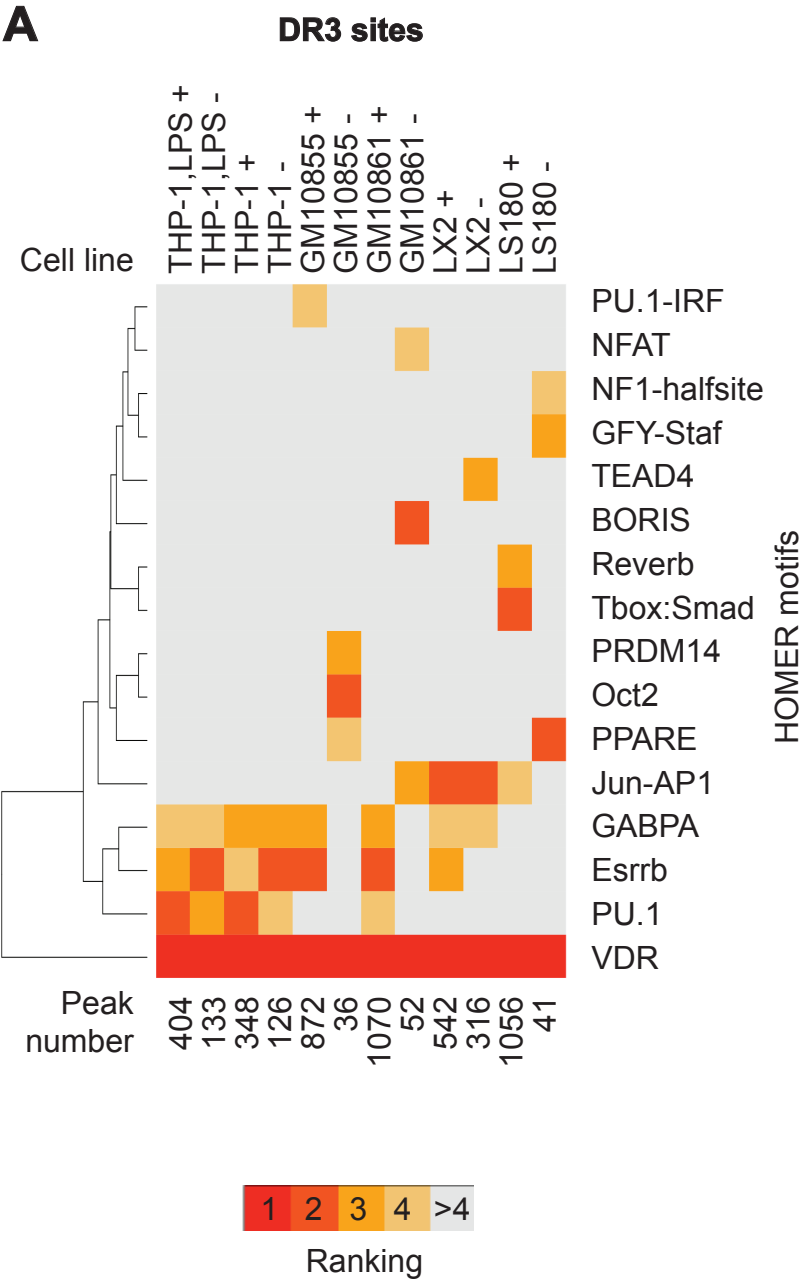

B

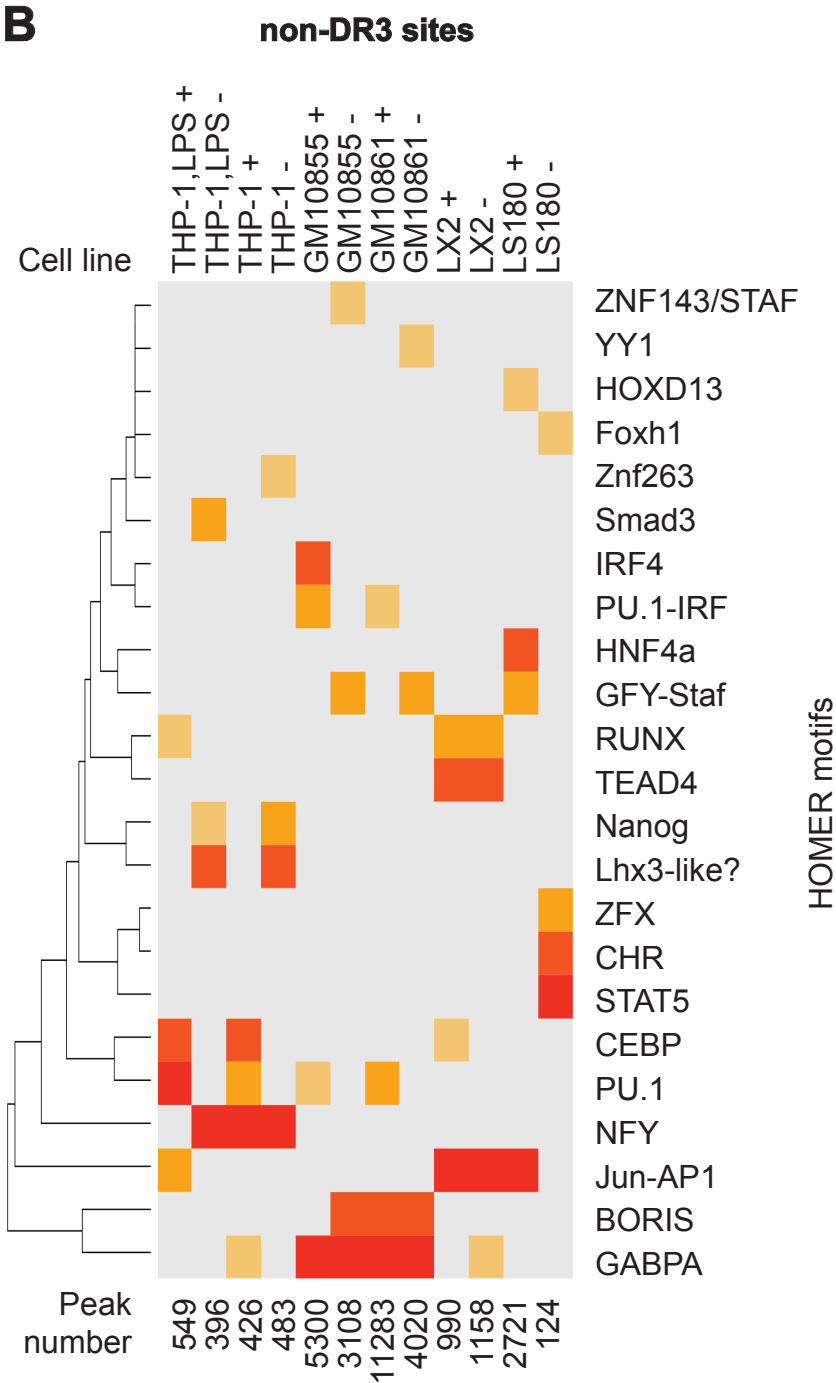

Supplement: Figure S6 — The occurrence of known transcription factor binding motifs below VDR ChIP-seq peaks. Known binding motifs were screened in the ±100 bp VDR ChIP-seq peak sets with (A) or without (B) the representative de novo DR3-type motif, ranked according to the significance of motif enrichment, subset to include only those that were within the top 4 in any sample, and displayed as hierarchically clustered heatmap of the ranks. Prior to screening, the known motif set supplied with the HOMER version 4.3 was reduced to remove similar motifs using a similarity cutoff 0.8 and replacing the HOMER-native DR3-type motif by the representative DR3-type motif from this study, i.e. that from stimulated, undifferentiated THP-1 cells. The numbers of screened peaks are given below the heatmaps for each sample. The + and - sign after the sample names on top indicate, respectively, whether the sample was ligand stimulated or not. (PDF) [file pone.0096105.s006.pdf]
